# Enhanced Lipid Peroxidation and Platelet Activation as Potential Contributors to Increased Cardiovascular Risk in the Low‐HDL Phenotype

**DOI:** 10.1161/JAHA.113.000063

**Published:** 2013-04-24

**Authors:** Natale Vazzana, Antonina Ganci, Angelo Baldassare Cefalù, Stefano Lattanzio, Davide Noto, Nicole Santoro, Raoul Saggini, Luca Puccetti, Maurizio Averna, Giovanni Davì

**Affiliations:** 1 Department of Internal Medicine and Center of Excellence on Aging, “G. d'Annunzio” University of Chieti, Chieti, Italy (N.V., S.L., N.S., G.D.); 2 Department of Internal Medicine, University of Palermo, Palermo, Italy (A.G., A.B.C., D.N., M.A.); 3 Department of Neuroscience and Imaging, “G. d'Annunzio” University of Chieti, Chieti, Italy (R.S.); 4 Division of Hematology, Atherothrombosis Center, University of Siena, Siena, Italy (L.P.)

**Keywords:** exercise, HDL cholesterol, oxidative stress, platelet

## Abstract

**Background:**

Low high‐density lipoprotein (HDL) levels are major predictors of cardiovascular (CV) events, even in patients on statin treatment with low‐density lipoprotein (LDL) at target. In animal models HDLs protect LDL from oxidation and blunt platelet activation. Our study aimed to examine whether HDL levels are related to in vivo oxidative stress and platelet activation, as determinants of atherothrombosis.

**Methods and Results:**

Urinary 8‐iso‐PGF_2α_ and 11‐dehydro‐TXB_2_, in vivo markers of oxidative stress and platelet activation, respectively, were measured in 65 coronary heart disease (CHD) normocholesterolemic patients with HDL ≤35 mg/dL, and in 47 CHD patients with HDL >35 mg/dL. The 2 eicosanoids were also measured before and after an intensive exercise program in sedentary people (n=18) and before and after fenofibrate treatment in otherwise healthy subjects with low HDL (n=10). Patients with HDL ≤35 mg/dL showed significantly higher urinary 8‐iso‐PGF_2α_ (median [25th to 75th percentiles]: 289 [189 to 380] versus 216 [171 to 321] pg/mg creatinine, *P*=0.019) and 11‐dehydro‐TXB_2_ (563 [421 to 767] versus 372 [249 to 465] pg/mg creatinine, *P*=0.0001) than patients with higher HDL. A direct correlation was found between urinary 8‐iso‐PGF_2α_ and 11‐dehydro‐TXB_2_ in the entire group of patients (ρ=0.77, *P*<0.0001). HDL levels were inversely related to both 8‐iso‐PGF_2α_ (ρ=−0.32, *P*=0.001) and 11‐dehydro‐TXB_2_ (ρ=−0.52, *P*<0.0001). On multiple regression, only 8‐iso‐PGF_2α_ (β=0.68, *P*<0.0001) and HDL level (β=−0.29, *P*<0.0001) were associated with urinary 11‐dehydro‐TXB_2_ excretion, independent of sex, age, smoking, hypertension, diabetes, previous myocardial infarction, total cholesterol, LDL, and triglycerides. Both intensive exercise and fenofibrate treatment significantly reduced the 2 eicosanoids in healthy subjects, in parallel with an HDL increase.

**Conclusions:**

A low HDL phenotype, both in CHD patients and in healthy subjects, is associated with increased lipid peroxidation and platelet activation. These data provide novel insight into the mechanisms linking low HDL with increased CV risk.

## Introduction

High‐density lipoprotein (HDL) cholesterol is a major independent risk factor for coronary heart disease (CHD).^1^

A low level of HDL cholesterol (HDL‐C) is a powerful predictor of increased cardiovascular (CV) risk,^2^ and it remains a significant risk factor in people whose LDL cholesterol (LDL‐C) is reduced to very low levels.^3^ Indeed, HDL‐C levels are predictive of major CV events in patients treated with statins, both when HDL‐C is considered as a continuous variable and when subjects are stratified according to quintiles of HDL‐C level. Even among subjects with LDL‐C levels <70 mg/dL, those in the highest quintile of HDL‐C level are at less risk for major CV events than those in the lowest quintile.^3^ Furthermore, the ratio of LDL to HDL cholesterol is also highly predictive of the risk of major CV events.

In patients with established CV disease, residual CV risk persists despite the achievement of target LDL‐C levels with statin therapy. Among patients with atherosclerotic CV disease and LDL‐C levels <70 mg/dL, there is no incremental clinical benefit from the addition of niacin to statin therapy during a 36‐month follow‐up period, despite significant improvements in HDL–C and triglyceride levels.^4^ Similarly, therapy with the cholesterol ester transfer protein (CEPT) inhibitor torcetrapib, despite a 72% increase in HDL‐C levels, resulted in an increased risk of CV mortality and morbidity.^5^ However, the clinical failure of torcetrapib intervention trials has been related to “off‐target” effects on neurohumoral regulation of blood pressure and increased risk of arrhythmias. In addition, CEPT inhibition generates large amounts of large HDL particles that are not optimally functional.^6^

Interventions aimed at increasing HDL‐C levels prevent the progression of CHD.^7^ In fact, niacin therapy, by increasing HDL levels, causes a significant regression of carotid intima–media thickness when combined with a statin, as compared with ezetimibe.^8^ Interestingly, the incidence of major CV events is lower in the niacin group than in the ezetimibe group.^8^ Intensive statin regimen, either with atorvastatin or rosuvastatin, results in very low levels of LDL‐C as well as a slight increase in HDL. The 2 regimens are similar in their ability to limit progression or induce regression of coronary disease evaluated by intravascular ultrasonography.^9^

In addition to its cholesterol‐transporting properties, HDL favorably regulates endothelial cell phenotype, promoting the production of nitric oxide (NO) by upregulating endothelial NO synthase expression. Moreover, HDL's antithrombotic properties may be related to their abilities to attenuate the expression of tissue factor and selectins and to downregulate thrombin generation via the protein C pathway, thus directly and indirectly blunting platelet activation.^10^

HDL exhibits antioxidant activity by inhibition of LDL oxidation with a subsequent reduction of cellular uptake by the monocyte macrophage system.^11^ A normo‐triglyceridemic, low HDL‐C phenotype is characterized by elevated oxidative stress and HDL particles with attenuated antioxidant activity.^12^ Antioxidant activity of small dense HDL is deficient in type 2 diabetes, and it is linked to enhanced oxidative stress.^13^ The increased generation of reactive oxygen species may induce enhanced lipid peroxidation of cell‐membrane phospholipids or circulating LDL, leading to the increased generation of F2‐isoprostanes, a family of prostaglandin isomers produced from arachidonic acid by a mechanism catalyzed by free radicals.^14–15^ F2‐isoprostanes can modulate the activation of platelets induced by low levels of other agonists.^16^ The consistent relationship between the rates of formation of F2‐isoprostanes and thromboxane (TX) in several metabolic disorders^16^ suggests that TX‐dependent platelet activation may be mediated, at least in part, by enhanced lipid peroxidation.^17^ This vicious cycle may be downregulated by a successful weight‐loss program in obesity^18^ or by tight metabolic control obtained by insulin treatment in type 2 diabetes mellitus.^19^

We speculated that increased oxidative stress in CHD patients with a low‐HDL phenotype could induce enhanced generation of 8‐iso‐prostaglandin (PG)F_2α_ and other biologically active isoeicosanoids and that these compounds, in turn, could contribute to platelet activation in this setting.^17^ Therefore, in the present study, we set out to investigate whether 8‐iso‐PGF_2α_ formation is increased in CHD patients with lower HDL levels (≤35 mg/dL) when compared with CHD patients with higher HDL levels (>35 mg/dL) and no significant differences in the other known CV risk factors and whether it correlates with the rate of TXA_2_ biosynthesis. Moreover, we examined the effects of 2 interventions aimed at increasing HDL levels, that is, fenofibrate treatment and 8 weeks of a standardized aerobic program, in otherwise healthy subjects with a low‐HDL phenotype by assessing time‐related changes in urinary 8‐iso‐PGF_2α_ and in TX‐dependent platelet activation.

The results of the present study suggest that enhanced peroxidation of arachidonic acid to form biologically active isoprostanes may represent an important biochemical link between the low‐HDL phenotype and persistent platelet activation in this setting.

## Methods

### Design of the Studies

The first study was a cross‐sectional comparison of urinary F2‐isoprostane 8‐iso‐PGF_2α_ and 11‐dehydro‐TXB_2_ (a major enzymatic metabolite of TXA_2_) of 65 normocholestoremic CHD patients with HDL‐C levels ≤35 mg/dL (52 men, aged 62 [54 to 70] years) with 47 normocholestoremic CHD patients with HDL‐C levels >35 mg/dL (30 men, aged 65 [57 to 68] years). CHD incidents (including angina pectoris, exercise stress testing positive for ischemia, and acute myocardial infarction in the previous 24 months) were recorded from the outpatient database or from hospital discharge diagnoses. All subjects were attending the Lipid Clinics of the University of Palermo School of Medicine. CV risk factors such as smoking, hypertension, and diabetes were equally represented in the 2 groups (Table 1).

**Table 1.** Clinical Characteristics of CHD Patients

Variables	HDL‐C ≤35 mg/dL (n=65)	HDL‐C >35 mg/dL (n=47)	*P* Value*
Age, y	62 (54 to 70)	65 (57 to 68)	0.439
Male sex, n (%)	52 (80)	30 (64)	0.09
Total cholesterol, mg/dL	155 (133 to 180)	166 (152 to 182)	0.063
Triglycerides, mg/dL	84 (55 to 153)	85 (62 to 116)	0.874
HDL‐C, mg/dL	28 (25 to 32)	43 (39 to 47)	<0.001
LDL‐C, mg/dL	110 (89 to 122)	99 (87 to 113)	0.153
Cigarette smoking, n (%)	21 (32)	13 (20)	0.749
Arterial hypertension, n (%)	36 (55)	28 (43)	0.804
Type 2 diabetes, n (%)	26 (40)	14 (22)	0.36
Previous MI, n (%)	24 (37)	16 (25)	0.90

Continuous variables are reported as median (25th to 75th percentiles). CHD indicates coronary heart disease; HDL‐C, high‐density lipoprotein cholesterol; LDL‐C, low‐density lipoprotein cholesterol; MI, myocardial infarction.

* By Mann–Whitney, chi‐square, or Fisher exact tests, as appropriate.

None of the patients had taken any drugs known to affect lipid metabolism or platelet function for ≥2 weeks before the start of the study because they were unwilling to do so. Patients with renal insufficiency or proteinuria (by serum creatinine level and urinalysis), altered hepatic function (by liver enzymes), or alcohol abuse were excluded.

All subjects were studied as outpatients after a 12‐hour fast. Blood samples were obtained for the following measurements: total cholesterol, triglycerides, HDL cholesterol. Each patient performed a 12‐hour overnight urine collection immediately before blood sampling. Urine samples were supplemented with the antioxidant 4‐hydroxy‐tempo (1 mmol/L) and stored at −20°C until extraction.

The study was approved by the local Ethics Committee, and all patients gave their written informed consent to participate in the study.

A second study was performed to investigate the effects of regular high‐amount, high‐intensity aerobic exercise on in vivo lipid peroxidation and platelet activation in 18 healthy sedentary people with low HDL‐C levels. We evaluated whether changes in HDL‐C levels, induced by the exercise program, were related to changes in the 2 eicosanoids. Each participant completed an 8‐week standardized aerobic high‐amount, high‐intensity training program. Urinary 8‐iso‐PGF_2α_ and 11‐dehydro‐TXB_2_ excretion rates had been measured before and after this intervention.

After giving written informed consent, 18 subjects (13 men, aged 50 [48 to 66] years) had been enrolled in the study. Subjects were enrolled if they had a sedentary lifestyle (regular aerobic exercise <3 times/weeks and <20 minutes/session), had a sedentary occupation, and had a baseline HDL cholesterol concentration <55 mg/dL (<1.4 mmol/L). Exclusion criteria included obesity (body mass index [BMI] ≥30 kg/m^2^), diagnosis of diabetes mellitus, poorly controlled hypertension or hypercholesterolemia, pregnancy, impaired liver or renal function, previous vascular event (myocardial infarction, stroke, transient ischemic attack), or other medical conditions that would preclude vigorous exercise, and treatment with nonsteroidal anti‐inflammatory drugs (NSAIDs), antioxidant supplements, anticoagulants, or antiplatelet drugs.

Each participant completed an 8‐week standardized aerobic training program. The exercise training involved 2 sessions per week of supervised exercise on a cycle ergometer (Monark 915E, Vansbro, Sweden). The exercise prescription in the exercise group was high‐amount, high‐intensity exercise for 55 minutes per session, the caloric equivalent of jogging ≈20 miles (32.0 km) per week at 60% to 75% of peak oxygen consumption.^20^ There was an initial period of 1 month during which the amount and intensity of exercise were gradually increased, followed by 8 weeks at the appropriate exercise prescription. Participants started at 55% of their baseline VO_2_ max for 45 minutes per session and progressed in intensity or duration every week according to a standardized protocol until achieving the standards scheduled for the training program (55 minutes at 75% of baseline VO_2_ max). All exercise sessions were verified by direct supervision or by heart rate monitors that provided recorded data (Polar Electro). Daily energetic consumption was monitored by metabolic armband (Sensewear Pro3).

A third study was performed to investigate the effects of 3‐month administration of fenofibrate (145 mg/day) to 10 subjects (7 men, aged 58 [55 to 60] years) with a low‐HDL phenotype and triglycerides >200 mg/dL, otherwise healthy, previously unresponsive to an appropriate dietary regimen (checked regularly by a qualified nutritionist). The 10 subjects were allocated to continue the previous diet plus fenofibrate for 3 months.

All patients had a positive familial history for CV events younger than age 65. Exclusion criteria included obesity (BMI ≥30 kg/m^2^), diabetes mellitus, poorly controlled hypertension or hypercholesterolemia, pregnancy, impaired liver or renal function, previous vascular event (myocardial infarction, stroke, transient ischemic attack), and treatment with NSAIDs, antioxidant supplements, anticoagulants, or antiplatelet drugs.

This study was approved by the local Ethics Committee, and all patients gave their written informed consent to participate in the study.

### Assays

All participants were instructed to perform an overnight urine collection and underwent a fasting blood sample drawn the following morning. Plasma, serum, and urine were stored in aliquots at −20°C until used for the various analyses.

Urinary 8‐iso‐PGF_2α_ and 11‐dehydro‐TXB_2_ excretion rates were measured by previously described radioimmunoassay methods.^21–22^ These methods have been validated by comparison with gas chromatography/mass spectrometry, as detailed elsewhere.^21–22^

Total cholesterol, triglycerides, high‐density lipoprotein cholesterol (HDL‐C), and low‐density lipoprotein cholesterol (LDL‐C) concentrations were measured as previously described.^23^

### Statistical Analysis

The Kolmogorov–Smirnov test was used to determine whether each variable had a normal distribution. When necessary, log‐transformation was used to normalize the data, or appropriate nonparametric tests were used. Comparisons of baseline data between the groups were performed by chi‐square statistics, Fisher exact tests, or Mann–Whitney *U* tests.

The differences between baseline and posttreatment values were analyzed with the Wilcoxon signed rank test. The association of eicosanoid measurements with other biochemical parameters was assessed by the Spearman rank correlation test.

Stepwise multiple linear regression analysis was performed to assess variables independently associated with urinary 11‐dehydro‐TXB_2_ excretion rates, with logarithmically transformed data for this analysis and forward selection of variables. Covariates included in the multiple regression models were selected on the basis of their significance in univariate analysis and their clinical relevance to the outcome of interest, as reported from other studies. They included sex, age, smoking, hypertension, diabetes, previous myocardial infarction, total cholesterol, LDL‐C, HDL‐C, triglycerides, and urinary 8‐iso‐PGF_2α_.

All values are reported as median (interquartile range [IQR]). *P* values <0.05 were regarded as statistically significant. All tests were 2‐tailed, and analyses were performed using the SPSS (version 16.0; APSS, Chicago, IL), statistical package.

## Results

The baseline characteristics of the 112 CHD patients are detailed in Table 1. The 2 groups of patients (CHD with HDL‐C levels ≤35 and >35 mg/dL) were comparable both for sex distribution and for age. No statistically significant differences were found between the 2 groups of patients for CV risk factors.

**Figure 1.**
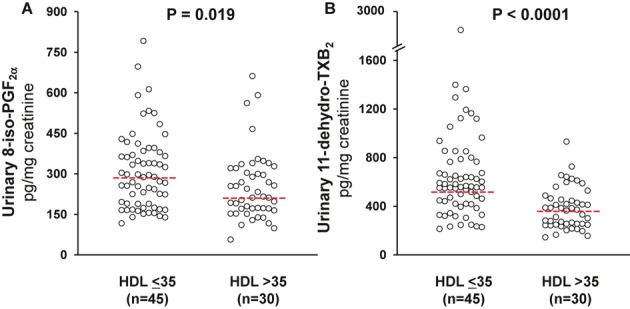
Urinary levels of 8‐iso‐PGF_2α_ (A) and 11‐dehydro‐TXB_2_ (B) in normocholesterolemic CHD patients, according to HDL cholesterol levels. The horizontal dotted lines denote the median value for each study group. 8‐iso‐PGF_2α_ indicates 8‐iso‐prostaglandin F_2α_; 11‐dehydro‐TXB_2_, 11‐dehydrothromboxane B_2_; CHD, coronary heart disease; HDL, high‐density lipoprotein.

Urinary excretion rate of the F2‐isoprostane 8‐iso‐PGF_2α_ was significantly (*P*=0.019) higher in the CHD patients with HDL ≤35 mg/dL than in the patients with higher HDL levels: 289 (189 to 380) versus 216 (171 to 321) pg/mg creatinine (Figure 1A). Moreover, patients with HDL ≤35 mg/dL had a significantly (*P*=0.0001) higher excretion rate of 11‐dehydro‐TXB_2_, an index of in vivo platelet activation, than did patients with higher HDL levels: 563 (421 to 767) versus 372 (249 to 465) pg/mg creatinine (Figure 1B).

**Figure 2.**
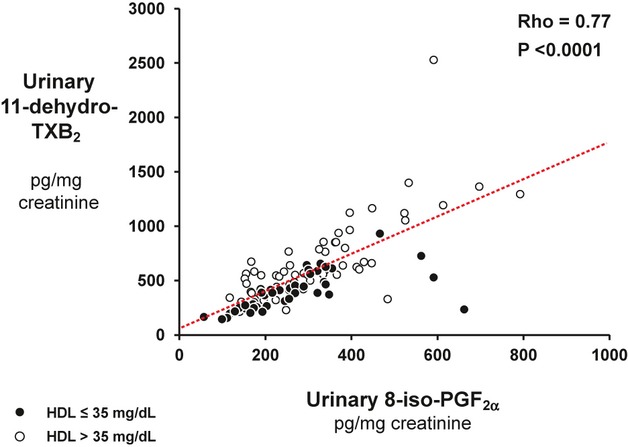
Correlation between urinary 8‐iso‐PGF_2α_ and 11‐dehydro‐TXB_2_ in CHD patients with HDL cholesterol ≤35 mg/dL (closed circles) and with HDL >35 mg/dL (open circles). 8‐iso‐PGF_2α_ indicates 8‐iso‐prostaglandin F_2α_; 11‐dehydro‐TXB_2_, 11‐dehydrothromboxane B_2_; CHD, coronary heart disease; HDL, high‐density lipoprotein.

**Figure 3.**
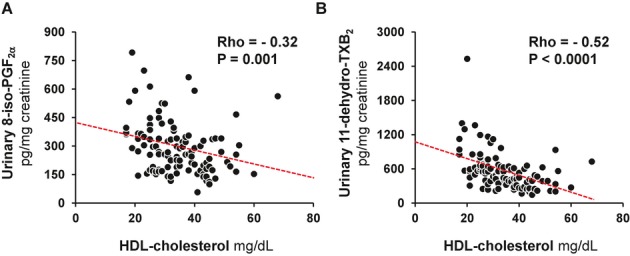
Correlations between HDL cholesterol and urinary 8‐iso‐PGF_2α_ (A) and 11‐dehydro‐TXB_2_ (B) in CHD patients. HDL indicates high‐density lipoprotein; 8‐iso‐PGF_2α_, 8‐iso‐prostaglandin F_2α_; 11‐dehydro‐TXB_2_, 11‐dehydrothromboxane B_2_; CHD, coronary heart disease.

As depicted in Figure 2, a highly statistically significant direct correlation was found between the rates of excretion of 8‐iso‐PGF_2α_ and 11‐dehydro‐TXB_2_ in both groups of patients (ρ=0.77, *P*<0.0001), suggesting a potential link between lipid peroxidation and platelet activation in this setting. In the entire group of CHD patients, HDL level was inversely related to both 8‐iso‐PGF_2α_ (ρ=−0.32, *P*=0.001) and 11‐dehydro‐TXB_2_ (ρ=−0.52, *P*<0.0001); see Figure****3.

On multiple regression analysis, only urinary 8‐iso‐PGF_2α_ (β=0.68, *t=*10.6, *P*<0.0001) and HDL level (β=−0.29, *t=*−4.56, *P*<0.0001) were associated with urinary 11‐dehydro‐TXB_2_ levels, independently of sex, age, smoking, hypertension, diabetes, previous myocardial infarction, total cholesterol, LDL‐C, and triglycerides.

### Effects of the Exercise Program

We investigated the effects of an 8‐week standardized high‐amount, high‐intensity aerobic exercise on in vivo lipid peroxidation and platelet activation in 18 healthy sedentary people with low HDL cholesterol levels. Because weight loss may significantly affect in vivo lipid peroxidation and platelet activation,^18^ subjects were given the recommendation of maintaining body weight during the 2‐month period, and thus they had a small but not significant weight gain.

Exercise training had no significant effect on total cholesterol or LDL‐C concentrations. There was a statistically significant increase (*P*=0.041) of HDL‐C levels, from 47.5 (42.5 to 49.0) to 52.5 (45.0 to 61.3) mg/dL, after the high‐amount, high‐intensity training period. There was also a significant reduction in triglyceride concentration (from 119 [92 to 177] to 95 [77 to 118] mg/dL, *P*=0.025).

**Figure 4.**
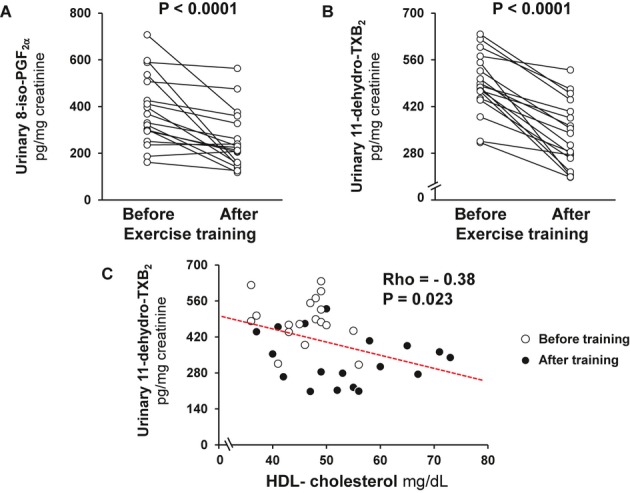
Urinary levels of 8‐iso‐PGF_2α_ (A) and 11‐dehydro‐TXB_2_ (B) in 18 healthy sedentary subjects before and after an aerobic high‐amount, high‐intensity training program. C, Correlation between urinary 8‐iso‐PGF_2α_ and 11‐dehydro‐TXB_2_ before (open circles) and after (closed circles) exercise training. 8‐iso‐PGF_2α_ indicates 8‐iso‐prostaglandin F_2α_; 11‐dehydro‐TXB_2_, 11‐dehydrothromboxane B_2_; HDL, high‐density lipoprotein.

The 8‐week exercise program was associated with a significant reduction in the urinary excretion rate of both 8‐iso‐PGF_2α_ and 11‐dehydro‐TXB_2_ (by 25% [9.3% to 58%] and 29% [20% to 40%], respectively, versus baseline; *P*<0.0001; Figure 4A and 4B).

Statistically significant inverse correlations were found between HDL‐C and 11‐dehydro‐TXB_2_ levels (ρ=−0.38, *P*=0.023) over the 8‐week training period by pooling the data obtained before and after exercise (Figure 4C).

### Effects of Fenofibrate

We next examined the effects of 3 months of administration of fenofibrate (145 mg/day) on the urinary excretion of 8‐iso‐PGF_2α_ and 11‐dehydro‐TXB_2_ in subjects with a low‐HDL phenotype and triglycerides >200 mg/dL but otherwise healthy to test the hypothesis of a cause–effect relationship between a low‐HDL phenotype and enhanced lipid peroxidation and platelet activation in the setting of healthy people with low HDL‐C levels.

**Figure 5.**
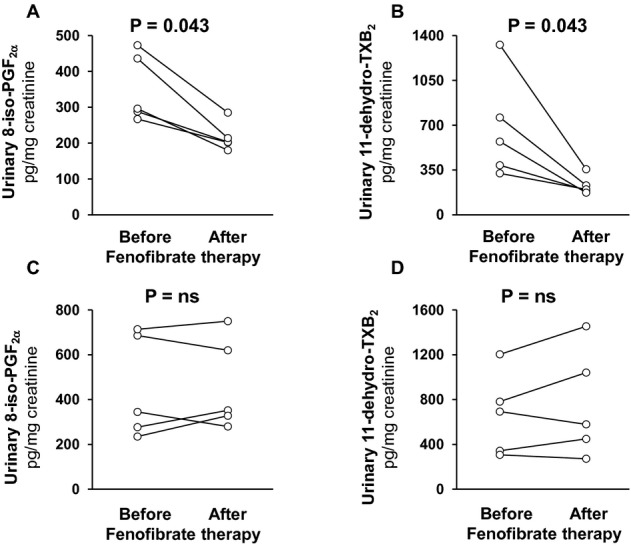
Urinary levels of 8‐iso‐PGF_2α_ and 11‐dehydro‐TXB_2_ in 10 healthy individuals with low HDL cholesterol and hypertriglyceridemia before and after a 3‐month fenofibrate (145 mg/day) treatment. A and B, Subjects who had an increase in HDL cholesterol above the median. C and D, Subjects who had an increase in HDL cholesterol below the median. 8‐iso‐PGF_2α_ indicates 8‐iso‐prostaglandin F_2α_; 11‐dehydro‐TXB_2_, 11‐dehydrothromboxane B_2_; ns, not significant; HDL, high‐density lipoprotein.

After fenofibrate treatment, HDL‐C levels were significantly increased, by 14% (5.0% to 25%; *P*=0.007), whereas triglyceride levels were significantly decreased, by 41% (38% to 46%; *P*=0.005). Despite this, both urinary 8‐iso‐PGF_2α_ and 11‐dehydro‐TXB_2_ excretion rates were not significantly changed in the whole group. However, in the 5 subjects in whom fenofibrate induced an increase of HDL cholesterol level above the median, we observed a significant reduction in urinary 8‐iso‐PGF_2α_ and 11‐dehydro‐TXB_2_ excretion rates, by 39% (27% to 45%) and 70% (45% to 72%), respectively (*P*=0.043) in comparison with no significant modifications obtained in the 5 subjects who had an increase of HDL cholesterol below the median (Figure****5). Moreover, in the former group of subjects, all individual values of urinary 8‐iso‐PGF_2α_ and 11‐dehydro‐TXB_2_ levels fell within the range of those of healthy subjects.^18^ Finally, the changes in 8‐iso‐PGF_2α_ levels were also associated with the reduction in urinary 11‐dehydro‐TXB_2_ levels because a statistically significant correlation was found between the rates of excretion of these eicosanoids throughout the range of values measured at baseline and after 3 months of administration of 145 mg of fenofibrate (ρ=0.77, *P*=0.009).

## Discussion

Low HDL‐C is an independent CV risk factor, and the increase of HDL‐C of only 1 mg/dL leads to a risk reduction of 2% to 3%.^1–2^ Despite aggressive LDL‐C lowering with statin therapy, a residual risk of CV morbidity and mortality still occurs in a significant portion of subjects.^3–4^ There is compelling evidence in animal models that increasing HDL levels is antiatherogenic.^1^ Therefore, raising HDL‐C was thought of as a strategy for reducing the residual CV risk. However, a recent systematic review and meta‐regression analysis of randomized controlled trials^24^ has suggested that increasing the amount of HDL‐C does not reduce the risk of coronary heart disease events and mortality.

HDLs have several protective properties that are independent of their involvement in cholesterol metabolism. They have antiatherogenic properties that reduce oxidation and vascular inflammation and improve endothelial function, thus promoting endothelial repair. Among antioxidant properties of HDL, the transfer of oxidized lipids from LDL to HDL, including oxidized phospholipids such as hydroperoxides and F2‐isoprostanes, facilitates subsequent degradation of these products by HDL enzymes or by delivery to the liver for degradation, thus preventing pathological activities of these molecules. Normal functional HDL has high anti‐oxidant potential, but when these antioxidant functions are overwhelmed by pathological processes, HDL is converted into a dysfunctional particle characterized by decreased anti‐inflammatory and antioxidant properties.^25^

In vitro studies show that HDL inhibits agonist‐stimulated platelet aggregation.^26^ This inhibitory effect is mediated by scavenger receptor type B1 and/or the apolipoprotein E receptor apoER2/LRP8 and is linked to the induction of intracellular signaling cascades encompassing stimulation of protein kinase C, cytoplasmatic alkalization, and generation of nitric oxide. Moreover, the infusion of Apo A‐1 Milano (a protective variant of the major protein constituent of HDL) into rats inhibits platelet aggregation.^10^

However, no evidence has been provided for the association between a low‐HDL phenotype and in vivo platelet activation as well as in vivo lipid peroxidation.

Biochemical evidence of increased platelet activation in vivo was obtained through noninvasive measurements of TX metabolite excretion^22,27^ that avoided artifactual platelet activation during and after blood sampling. Although we did not measure platelet aggregation in our study, it should be emphasized that the *ex vivo* measurement of platelet responses to various agonists represents an index of functional capacity that by no means reflects the extent of platelet activation in vivo.^27^ The excretion rate of the major enzymatic metabolite of TXA_2_, 11‐dehydro‐TXB_2_, has been validated as a reliable and noninvasive, integrated index of in vivo platelet activation.^17^

Moreover, increased generation of reactive oxidant species can induce enhanced lipid peroxidation of cell‐membrane phospholipids or circulating LDL, leading to increased generation of F2‐isoprostanes, bioactive compounds produced from arachidonic acid by a free radical–catalyzed mechanism of lipid peroxidation.^28^ Activated platelets are also a source of reactive oxidant species (by NADPH oxidase)^29^ and might contribute to isoprostane generation by COX‐dependent mechanisms.^30^ It has been proposed that F2‐isoprostanes might transduce the effects of oxidant stress associate with complex metabolic disorders into specialized forms of cellular activation. In particular, 8‐iso‐PGF_2α_ might be able to modify platelet adhesive reactions and platelet activation in vitro by low concentrations of agonists.^16^

In the present report, we performed a series of studies to test the hypothesis that persistent in vivo platelet activation maybe related, at least in part, to enhanced urinary excretion of the F2‐isoprostane 8‐iso‐PGF_2α_ as a marker of in vivo lipid peroxidation in the setting of a low‐HDL phenotype.

In the first study, we performed a cross‐sectional evaluation of the 2 eicosanoids in normocholesterolemic CHD patients, and we found that the formation and urinary excretion of 8‐iso‐PGF_2α_ was elevated in the vast majority of a relatively large group of patients with HDL‐C levels <35 mg/dL in comparison with a group of CHD patients with higher HDL‐C levels, carefully characterized for other variables potentially influencing lipid peroxidation (see Table 1).^28^ Moreover, we have identified a novel mechanism through which low HDL‐C levels may affect CV morbidity and mortality, that is, TX‐dependent platelet activation. In fact, low HDL‐C levels in CHD patients were associated with a significantly higher rate of TX metabolite excretion than measured in CHD patients with higher HDL‐C levels. The consistent linear relationship between the excretion of rates of 8‐iso‐PGF_2α_ and 11‐dehydro‐TXB_2_ demonstrated in our study confirms and extends previous findings in subjects with other CV risk factors such diabetes mellitus or hypercholesterolemia.^17^

Further evidence for a cause‐and‐effect relationship between low HDL‐C level and persistent platelet activation was obtained through 2 interventional studies: the first in sedentary people, the second in subjects with low HDL‐C levels but otherwise healthy.

When we performed a study with a high‐intensity, high‐amount exercise program for 2 months for sedentary people, we observed significant decreases in both TX‐dependent platelet activation (11‐dehydro‐TXB_2_) and lipid peroxidation (8‐iso‐PGF_2α_), with an inverse correlation between the TX metabolite and HDL level. Our data are in accordance with a recent study of adolescents with metabolic syndrome reporting that a physical activity program induced amelioration of HDL levels as well as of markers of oxidative stress.^31^

In healthy subjects with low HDL‐C and high triglycerides, the reduction in both lipid peroxidation and TX‐dependent platelet activation was proportional to the effect of fenofibrate on HDL levels. In fact, HDL‐C increase above the median led to normalization of the 2 noninvasive indexes of platelet activation and lipid peroxidation. This is consistent with the finding that age‐related changes in middle‐aged nonobese men show a simultaneous decrease in HDL‐C levels as well as plasma oxidative stress markers (8‐iso‐PGF_2α_ and malondialdehyde).^32^

The results of our study suggest that markers of lipid peroxidation and TX‐dependent platelet activation may be useful synergistically with measurement of dysfunctional HDLs to improve CV risk stratification in patients with low HDL‐C.

Several limitations of the intervention studies should be acknowledged. These include lack of randomization and small sample size of the 2 interventional studies. Despite these limitations, our findings may have important clinical implications for primary and secondary prevention in patients with a low HDL phenotype, providing novel insight into the mechanisms linking low HDL and occurrence of CV disease. In fact, within the limits of our relatively small mechanistic studies with biochemical end points, our results suggest that a substantial reduction in TX‐dependent platelet activation can be achieved by a significant increase in HDL levels obtained through different tools (exercise and fenofibrate). For subjects who fail to achieve a substantial HDL‐C increase, low‐dose aspirin may be considered as an option after evaluating the potential benefit and hemorrhagic risk of the individual patient.^33^
